# Implementation of the Health System Reform Plan in Hospital Emergencies of Iran: A Qualitative Study

**Published:** 2020-09-23

**Authors:** Shahriar Janbazi, Ayad Bahadorimonfared, Mostafa Rezaei-Tavirani, Ali Maher, Mojtaba Zonoobi, Naser Vazifehshenas, Khatereh Hanani

**Affiliations:** 1 Faculty of Medicine, Shahid Beheshti University of Medical Sciences, Tehran, Iran.; 2 Department of Health & Community Medicine, Faculty of Medicine, Shahid Beheshti University of Medical Sciences, Tehran, Iran.; 3 Proteomics Research Center, Faculty of Paramedical Sciences, Shahid Beheshti University of Medical Sciences, Tehran, Iran.; 4 Department of Health Policy, Economics & Management, School of Management and Medical Education, Shahid Beheshti University of Medical Sciences, Tehran, Iran.; 5 Information Technology Unit, Shahid Beheshti University of Medical Sciences, Tehran, Iran.; 6 Resource Management, Central Library & Archives, Shahid Beheshti University of Medical Sciences, Tehran, Iran.; 7 Statistics & Information Technology Management, Shahid Beheshti University of Medical Sciences, Tehran, Iran.

**Keywords:** Health Care Reform, health policy, Emergency Service, Hospital, Outcome Assessment, Health Care, Health Plan Implementation

## Abstract

**Introduction:**

Health and efforts to maintain and promote it have always been an essential priority in various countries. This study aims to evaluate the implementation of the health system reform plan in emergency departments in Iran.

**Methods:**

This qualitative study evaluated five dimensions (finance, social responsibility, growth and learning, service recipients, and internal processes) through examining 70 indicators in 400 healthcare service providers and 300 healthcare recipients after the implementation of reform plan in ten emergency departments affiliated to Shahid Beheshti University of Medical Sciences using the balanced scorecard based on comprehensive evaluation model for the health system reform plan in Iran (CEHSRP-IR), from 2018 to 2019.

**Results:**

From the perspective of 51% of service providers and 55% of service recipients, the health system reform plan has achieved its goals in hospital emergencies. Significant gap between the ideal effectiveness and the current situation in health services in hospital emergencies was observed, especially in the educational and financial dimension.

**Conclusion:**

According to the findings of this study, adaptation of activities and programs to the model proposed for emergency departments in Health Reform Plan is essential for improving its effectiveness.

## Introduction

 The Ministry of Health and Medical Education of Iran has proposed a plan to reform the health system with eight executive packages aimed at reducing the payment of hospitalized patients in public hospitals, supporting the physicians to stay in deprived areas, planning for specialist physicians to be present in public hospitals, improving the quality of hoteling in public hospitals, promoting natural childbirth program, and providing financial protection for incurable unique diseases, and patients in need since 05/05/2013. The next phase of this plan started after a while in the field of health (1). 

 Today, patient’s satisfaction with the treatment sector is recognized as a critical indicator of the efficiency of the corresponding organization (2). On the other hand, patient satisfaction is an essential indicator of emergency care quality (3). Patient satisfaction can be defined as the recipient's response to the services provided, which reflects his or her overall understanding of the quality of service provided (4). How the service is provided in this ward can reflect the general state of service delivery throughout the hospital. In general, due to the emergency unit's importance, increasing satisfaction in this ward has a significant effect on people's attitudes towards the hospital (5). With the introduction of new management concepts to the medical world, today, emergency departments are concerned with patient satisfaction and rights more than ever.

In contrast, the emergency department faces more challenges compared to other parts of the hospital, which can reduce patient satisfaction (6). The quality of service is crucial in evaluating the health sector, especially hospitals, and to evaluate it accurately and effectively, a "scientific standard and model" is needed to compare quantitative and qualitative performance with standards so that we can find out how the situation is and take action to solve its problems. Due to the need for quantitative and qualitative improvement of services in the hospital emergency department, this area is of particular importance and requires proper knowledge (7).

**Figure1:**
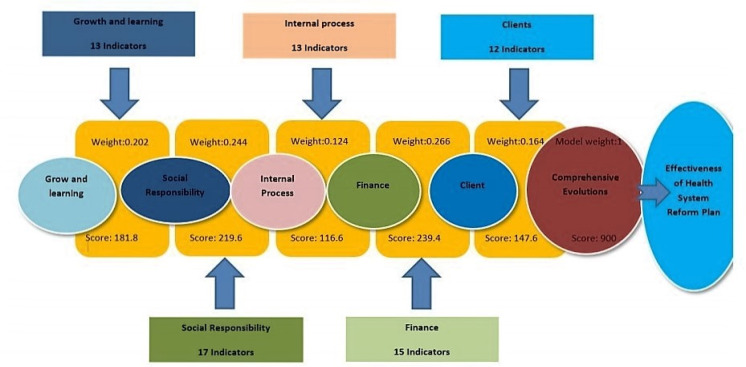
Comprehensive Evaluation Model for Health System Reform Plan in Iran (Janbazi et al., (13); this figure is used with permission).

 In an applied study, a comprehensive model was designed to evaluate the health system transformation plan in Iran. The feature distinguishing this study from previous studies is the simultaneous attention to performance appraisal challenges, social responsibilities, and study in a comprehensive and coherent manner. The 900-balanced scorecard based on comprehensive evaluation model of the health system reform plan in Iran is based on five dimensions (finance, social responsibility, growth and learning, clients, and internal processes), 17 components, and 70 indicators. This comprehensive model is called CEHSRP-IR for short. It has a comprehensive approach in four levels (policymakers and strategic managers, resource providers, service providers, and service recipients) and uses qualitative and quantitative research methods (e.g., Content analysis, pairwise comparisons, Delphi method), and is an accurate tool for practical evaluation of the health reform plan (Figure 1). This study aimed to evaluate the implementation of the health system reform plan in emergency departments in Iran using the mentioned tool. 

## Methods

***Study design and setting***

This is a qualitative study conducted in 2018-2019. The health system transformation plan was evaluated in ten emergency departments of hospitals (educational and non-educational) affiliated to Shahid Beheshti University of Medical Sciences using the comprehensive evaluation model of the health system reform plan in Iran (CEHSRP-IR). Five educational hospitals (including Imam Hossein, Loghman Hakim, Shohadaye Tajrish, Modarres, and Taleghani) and five non-educational hospitals (including Imam Khomeini of Firoozkooh, Sevvom-e-Shaban of Damavand, Shohadaye Gomnam, Zaeem Pakdasht, and Shohadaye Pakdasht) were studied. In all stages of collecting and analyzing the data of this research, ethical considerations, observance of data integrity, and confidentiality of the participants' opinions in the evaluation have been observed. Besides, this study has been approved by the National Ethics Committee with the ethics code of IR.SBMU.RETECH.REC.1397.1394, to observe ethical considerations and confidentiality.

The present study was conducted based on a matrix that includes five dimensions (learning and growth, internal processes, finance, clients, social responsibilities), four levels (service providers, service recipients, resource providers, and managers), 70 indicators, and 900-balanced scorecard model. In this model, the center with the highest score has been the most effective in achieving the goals defined in the five dimensions and, ultimately, the best efficiency in implementing the health system reform plan. 

 In this study, based on the sample size calculated using Cochran's formula, 400 staff members from the emergency departments of the ten hospitals were randomly selected for evaluating the implementation of the health system reform plan.

 This study's practical purpose was to use the CEHSRP-IR model to evaluate the ten emergency departments in terms of adherence to the health system reform plan. In addition, the viewpoint of emergency service recipients (patients) regarding the implementation of the health reform plan was evaluated in two hospitals, which had the highest and lowest evaluation scores in the first phase, using this model. Shahid Beheshti University of Medical Sciences, covers about 52% of Tehran's population, which was selected to implement the health system reform plan. The population covered by this university alone is more than 19 provinces of the country, such as Golestan, Yazd, Hamedan, Qazvin, Qom, and Kermanshah (8). 

***Participants***

The participants of the study, which was performed in two phases, consisted of the treatment staff (service provider) and patients who referred to the emergency departments of the studied hospitals. In the first phase, the sample size was estimated as 400 participants, and in the second phase, it was estimated as 300 participants using Cochran's formula. In the first phase, the samples were selected via target stratified random sampling and in the second phase using a simple random sample. 

***Data gathering and analysis***

The effectiveness of the emergency department of the studied hospitals was measured from the perspectives of both the service providers and the service recipients using a standard questionnaire that was designed based on the model (CEHSRP-IR), and its validity and reliability were confirmed. Second-order confirmatory factor analysis has been used to confirm this model. Therefore, Smart PLS software was used to perform this analysis. According to the analysis outputs, all obtained factors’ load coefficients were greater than 0.4. Also, Cronbach's alpha and reliability in this model were higher than 0.7. Then, to measure the composite reliability, the CR (Composite Reliability) statistic was examined, which was higher than 0.7. This indicates the proper fit of the model. Significance coefficients of Z were examined to measure the good-fitting structural model, the value of which was more than 1.96 for all obtained coefficients, and shows the significance of all questions and relationships between variables with 95% confidence level. In the R Squares criterion, all values related to the endogenous variables of the model were greater than 0.4, which shows a strong fit of the structural model. Data were then analyzed using software including the office version, 2019 and SPSS version, 2017, and specialized CEHSRP-IR software (ver2). 

## Results

***3.1. Evaluation from the perspective of service providers***

     In this phase, the standard questionnaire consisting of 70 items from the indicators in the model (CEHSRP-IR), was given to 400 emergency staff from five educational hospitals (including Imam Hossein, Loghman Hakim, Shohadaye Tajrish, Modarres, and Taleghani) and five non-educational hospitals (including Imam Khomeini of Firoozkooh, Sevvome Shaban of Damavand, Shohadaye Gomnam, Zaeem Pakdasht, and Shohadaye Pakdasht). Results are presented below: 

***3.1.1 General evaluation***

The function of the evaluation model used in this study, which rates the effectiveness of the performance of health service providers based on their score, is to identify their strengths and weaknesses in a general and specialized way (separately for the five dimensions of the model). First, a general evaluation of the performance of the studied hospital emergencies was performed, the results of which are as follows:

Based on the evaluation on the 900-balanced scorecard model (CEHSRP-IR), the emergency department of Shahid Modarres Hospital ranked first in implementation of the health system reform plan with 554.38 points and Zaeem Pakdasht Hospital with 364.17 points ranked last (tenth) (Table 1). Findings of this stage showed that the emergency department of Shahid Modarres Hospital, despite being 345.62 points away from the ideal point of effectiveness of the health system, had the highest rank among the ten centers studied. Its difference with Zaeem Pakdasht Hospital, which was at the bottom of the ranking of this study, is a positive distance of 190.21 points.

***3.2 Evaluation of five dimensions***

As shown in Table 1, the rankings of the studied hospital emergency departments in each dimension were determined based on the comprehensive evaluation model. Therefore, each hospital's emergency department must eliminate its weaknesses to improve the services provided to the people in the health reform plan. Using the analysis provided by the relevant software, the details of the points and strengths and weaknesses of each dimension can be seen in each center, and they lay grounds for plans to eliminate the shortcomings and effectively implement the health system reform plan.

***3.2.1 Evaluation of growth and learning***

According to the assessment made in the growth and learning dimension of the health system reform plan in the emergency room of the ten studied hospitals (Table 1), Shahid Modarres Hospital with a score of 135.18 and Taleghani Hospital with a score of 98.4, and Sevvom Shaban Hospital in Damavand with a score of 98.2 have the lowest ranks. It is important to note that Taleghani Hospital is an educational hospital, but has received a low score (98/4) in the evaluation, so it is recommended to pay special attention to strengthening the indicators of growth and learning in this hospital.

***3.2.2. Evaluation of social responsibilities***

According to the evaluation made in the social responsibility dimension of the health system reform plan in the emergency departments of the ten studied hospitals, as shown in Table 1, Shahid Modarres Hospital with a score of 136.97 ranked first and Zaeem Hospital in Pakdasht ranked last with a score of 82.93. Therefore, it is suggested to pay more attention to the social responsibilities that have a special place in the health system reform plan and its indicators in the hospitals that have not obtained the desired score.

***3.2.3. Evaluation of internal processes***

According to the results of the evaluation of the internal processes of the health system reform plan in the emergencies of the ten studied hospitals, as shown in Table 1, Shahid Modarres Hospital with a score of 56.09 and Sevvom-e- Shaban Hospital in Damavand with a score of 38.03 ranked first and last, respectively. Therefore, it is suggested that efforts be made to eliminate the shortcomings of internal processes and to modify and shorten the processes and eliminate redundant processes to improve the effectiveness of the health reform plan.

***3.2.4. Assessments of clients***

 The evaluation of the clients (patients) of the health system reform plan in the emergency departments of the 10 studied hospitals is shown in Table 1, Shahid Modarres Hospital, with 97.05 and Zaeem Hospital in Pakdasht with a score of 66.57 points ranked first and tenth, respectively. Therefore, it is suggested to attempt to improve the desired indicators in the client dimension to increase effectiveness and client satisfaction.

***3.2.5 Evaluation of financial dimension***

 According to the evaluation of the health system reform plan's financial dimension in the emergency departments of the ten studied hospitals, as shown in Table 1, Imam Hossein Hospital with a score of 158.88 and Zaeem Hospital in Pakdasht with a score of 86.08 are ranked first and tenth, respectively. It is suggested to plan to improve the desired indicators in the financial dimension to enhance the effectiveness of the health reform plan. Studies show that since Imam Hossein Hospital was the first hospital in the experimental establishment of the financial system related to the health system reform plan in the country, it has had more experience, knowledge, and acceptable growth in this model dimension.

***3.3. Evaluation from the perspective of service recipients***

According to the findings of this study in the previous section, it was decided to evaluate the health system reform plan in two hospitals of Shahid Modarres with a capacity of 370 beds and Zaeem Hospital in Pakdasht with a capacity of 120 beds, which had obtained the highest and lowest scores in the first phase of evaluation. In this phase, data were collected and analyzed using the opinions of 300 service recipients and using the standard questionnaire. The findings of this section show that 51% of the studied patients were satisfied with the hospital emergency services in providing health services. In this dimension, which has 160 points, Modares Hospital, with a score of 107.88, achieved the satisfaction of 67.50% of the clients. With a score of 55.90, Zaeem Hospital has not been able to keep more than 35% of the service recipients satisfied.

## Discussion

 The study's findings revealed that despite the high volume of work and increasing services provided to patients, the studied emergency departments still have a long way to reach the ideal point regarding the effectiveness of services from the viewpoint of the target community. The acquired score of the studied hospitals in the form of the comprehensive evaluation model used, especially, the average score obtained in the evaluation phase of service recipients' feedback, which indicates 55% satisfaction, indicates the need to review the health system reform plan.

Emergency service, as the first line for dealing with patients, provides medical services that can be presented in the form of a health reform plan. On the other hand, very little research has been done to evaluate Iran's health reform plan. Therefore, this research has the advantage that has evaluated the services provided in SBMU, the largest community providing healthcare based on the health system reform plan.

 A study conducted by Dehghan et al. (2016) intended to examine the performance of the health system reform plan from the perspective of Yazd university hospital executives, 47.2% of managers stated that the health system reform plan had achieved 60 to 79% of their goals. Also, 49.1% of managers believed that the health system's reform plan has been able to achieve a lot in dealing with out-of-tariff costs. Another belief of managers was that patients are more satisfied with the health system reform plan than doctors and nurses (9). However, the results of the present study do not match the findings of Dehghan et al., because the evaluation score of the reform plan in this study in the best hospital (Modarres) shows 61.6% success and in the worst evaluation score (Zaeem Pakdasht) is 40.46%. Besides, the evaluation results from patients' point of view, is not in agreement with the research findings of Dehghan et al.. It seems that four years after the implementation of the plan, the occurrence of defects and possible disruptions is one of the reasons for the decrease in inpatient and service providers' satisfaction.

 In a study conducted by Goodarzi et al. (2015), they concluded that patients were more satisfied with the health system reform plan than the medical and treatment staff. Conversely, nursing and support staff have been most dissatisfied with the health system reform plan. On the other hand, the satisfaction of health care service providers encourages hospital managers to the continue the medical services provided and their commitment, and the dissatisfaction of managers and employees has undesirable consequences on the plan. The dissatisfaction of managers and medical staff leads to their disconnection from the health care system or at least their non-participation in the provision of services (10). Therefore, the findings of this study showing the increase of dissatisfaction among both staff and patients with the health reform plan are inconsistent with the results of Goodarzi et al.'s research. In a study conducted by Sinaki et al. (2015), which analyzed job satisfaction of medical staff after the implementation of the health system reform plan in Kosar Hospital in Qazvin, the results showed that the overall job satisfaction of the majority of medical staff was at a moderate level (11). The findings of this study are consistent with the findings of Sinaki et al.

 In a study conducted by Wu et al. (2019) in which 216 professional staff rated the importance of the main views of the balanced scorecard, the ranks were as follows: patient, internal process, learning and growth, and finances. The weight-based analysis highlighted the importance of all indicators and areas that needed serious attention in future planning and management. This study recommended creating a balanced scorecard measurement system for integrated care organizations in China (12). The present study considered social responsibility in addition to the other dimensions addressed in in Wu's research. Regarding the prioritization of dimensions, the present study differs from Wu's research.

Based on the results of this research, in the learning and growth dimension, using health data in line with research goals and unraveling the country's health problems, reforming the educational system based on the country's health map, expanding creativity, innovation and technology in the field of health, and comprehensive knowledge management in all levels of the health transformation plan had the most significant weight and impact on the effectiveness of the plan and its outcome in hospital emergency services, respectively. 

According to the results of this research, in social responsibilities dimension, system establishment and participation of all groups involved in the health reform plan to actively monitor the implementation of the plan, full development of services including insurance and health services in all parts of the country for different social classes, the commitment of government agencies to comprehensively implementing the plan, and paying attention to the fundamental rights of the people had the most significant weight and impact on the effectiveness of performance and plan outcome, respectively. 

Based on the results of this research, in internal processes dimension, eliminating interference chains in the service path, especially emergencies, development of standard protocols, and use of intelligent systems, attracting the participation of institutions and organizations at the international, regional, national and local levels had the highest weight and impact on the effectiveness of the project, respectively. 

 According to the results of this research, in the financial dimension, intelligent and purposeful monitoring of supply, distribution and consumption of equipment and pharmaceutical and medical supplies, financing of the plan from public and charitable budgets, progress and effectiveness, and optimizing income, payments and investment based on service levels had the most significant weight and impact on the effectiveness of performance and outcome of the plan, respectively. 

 Based on the results of this study, in the service recipient dimension, maximum satisfaction of project clients, community health growth and disease management, and creating transparency platforms had the highest weight and impact on the effectiveness of the plan and outcome of the design, respectively. 

**Table1:** Ranking of emergency departments of Shahid Beheshti University of Medical Sciences based on the scores of health reform plan evaluation dimensions

**Hospitals**	**Evaluation dimensions**					**Total Score**
	**Growth &** **Learning**	**Social** **Responsibility **	**Internal** **Processes**	**Clients**	**Financial**	
Shahid Modarres	135.8	136.97	56.9	97.05	144.92	554.38
Imam Hossein	120.8	131.4	49.73	81.3	156.88	524.8
Loghman Hakim	131.4	129.57	54.1	80.88	117.93	495.17
Shohadaye Pakdasht	125.02	112.9	52.45	88.6	109.45	472.4
Imam Khomeini*	114.55	116.72	48.67	86.33	121.1	471.38
Shohadaye Gomnam	117.75	110.5	47.1	82.08	124.43	466.13
Shohadaye Tajrish	115.42	121.37	47.15	77.1	110.25	456.15
Sevvome Shaban**	98.2	106.27	38.03	73.63	109.95	413.12
Taleghani	98.4	102.48	41.3	74.2	100.28	404.43
Zaeem Pakdasht	101.13	82.93	39.9	66.57	86.8	364.17

*Firoozkooh; ** Damvand.

## Conclusion

Analysis of the results of this study and the average of study measurements showed that the health system reform plan has reached its goals from the perspective of 51% of service providers and 55% of service recipients in the emergency services of the studied hospitals. These results, together with the ranking result of the studied centers, indicate a significant distance between the effectiveness of the health system reform plan and the ideals in the evaluation model. This is consistent with the findings of most health researchers in Iran; thus, highlighting the significant gap between the results and the objectives of the project, which indicates the need for re-evaluating this national program. A huge amount of financial and human resources of the country has been spent on this program, but its efficiency has only been about 50%.
